# Response Rates for Patient-Reported Outcomes Using Web-Based Versus Paper Questionnaires: Comparison of Two Invitational Methods in Older Colorectal Cancer Patients

**DOI:** 10.2196/jmir.3741

**Published:** 2015-05-07

**Authors:** Nicole JE Horevoorts, Pauline AJ Vissers, Floortje Mols, Melissa SY Thong, Lonneke V van de Poll-Franse

**Affiliations:** ^1^Centre of Research on Psychology in Somatic Diseases (CoRPS)Tilburg UniversityTilburgNetherlands; ^2^Netherlands Comprehensive Cancer OrganisationUtrechtNetherlands

**Keywords:** Internet, questionnaires, aged, aged, 80 and over, cancer, colon, cancer, rectum, characteristics, population, survey methods, respondents, patient-reported outcomes

## Abstract

**Background:**

Improving questionnaire response rates is an everlasting issue for research. Today, the Internet can easily be used to collect data quickly. However, collecting data on the Internet can lead to biased samples because not everyone is able to access or use the Internet. The older population, for example, is much less likely to use the Internet. The Patient-Reported Outcomes Following Initial Treatment and Long-Term Evaluation of Survivorship (PROFILES) registry offers a platform to collect Web-based and paper questionnaires and to try different measures to improve response rates.

**Objective:**

In this study, our aim was to study the influence of two methods of invitation on the response rate. Our second aim was to examine the preference of questionnaire mode of administration (paper or Web-based) for the older patient in particular.

**Methods:**

To test these two invitational methods, 3406 colorectal cancer patients between ages 18 and 85 years received an invitation containing an access code for the Web-based questionnaire. They could also request a paper questionnaire with an included reply card (paper-optional group). In contrast, 179 randomly selected colorectal cancer patients received a paper questionnaire with the invitation (paper-included group). They could also choose to fill out the Web-based questionnaire with the included access code.

**Results:**

Response rates did not differ between the paper-optional and the paper-included groups (73.14%, 2491/3406 and 74.9%, 134/179, *P*=.57). In the paper-optional group, online response was significantly higher when compared to the paper-included group (41.23%, 1027/2491 vs 12.7%, 17/134, *P*<.001). The majority of online respondents responded after the first invitation (95.33%, 979/1027), which was significantly higher than the paper respondents (52.19%, 764/1464, *P*<.001). Respondents aged 70 years and older chose to fill out a paper questionnaire more often (71.0%, 677/954). In the oldest age group (≥80 years), 18.2% (61/336) of the respondents filled out a Web-based questionnaire.

**Conclusions:**

The lack of difference in response rates between invitation modes implies that researchers can leave out a paper questionnaire at invitation without lowering response rates. It may be preferable not to include a paper questionnaire because more respondents then will fill out a Web-based questionnaire, which will lead to faster available data. However, due to respondent preference, it is not likely that paper questionnaires can be left out completely in the near future.

## Introduction

The first Web-based questionnaires were posted in the mid-1990s, but they were only available for a select few with access to a computer and to the Internet [1]. Today, the Internet is accessible for more and more households. In the Netherlands, access to the Internet is high with 97% of the households having an Internet connection in 2013 [2]. To optimally utilize this high level of access, the population-based Patient-Reported Outcomes Following Initial treatment and Long-Term Evaluation of Survivorship (PROFILES) registry was developed in 2010. Its goal is to collect, preferably, online data on patient-reported outcomes (PRO) of cancer patients at least once a year [3]. However, paper questionnaires can be provided if preferred by the respondent. Offering different modes of administration is a way to improve response as is offering incentives and sending reminders [4].

Although it is widely accepted that Web-based questionnaires offer advantages, these advantages are not all scientifically proven. Advantages of Web-based questionnaires compared to paper questionnaires that are supported by literature are more complete data [5], less data entry errors [6], and questionnaires returned more quickly [7]. Several studies also show that reliability of Web-based questionnaires and paper questionnaires is comparable [8-11]. An often-described disadvantage of Web-based questionnaires is sample bias [1] because not all population groups have access to or are proficient in using the Internet. Additionally, Web-based questionnaires often have lower response rates than paper questionnaires [12].

Recent literature shows that the online respondent is most likely to be young and highly educated [10,13,14]. On the other hand, cancer patients tend to be older. There are a few studies that report on Internet use of older patients, but these patients are described as one group (eg, 50 years and older or 65 years and older) with response percentages varying from 58% to 63% [15-20]. Studies on computer and Internet use that are stratified by age and include a group older than 60 years are sparse. We found 6 studies that stratified older age into groups for computer or Internet use [21-26]. Percentages varied from 10% for those aged ≥85 years for Internet use in 2007 [22] to 35% in the 60-69 years age range for computer use for email in 2011 [21]. A previous study from our group, performed in 2007, observed Internet access for 3 age groups (<50 years, 50-59 years, and 60-69 years) of 81%, 65%, and 47%, respectively [24]. According to Statistics Netherlands, 54% of Dutch individuals between ages 65 and 75 years had access to the Internet in 2007 [27]. Since 2007, access to the Internet in the Netherlands has increased rapidly. In the older population aged 65-75 years, 80% currently have access to the Internet [28]. Therefore, we expect that more older respondents will be able to fill out Web-based questionnaires.

This paper describes a study in which our primary aim was to investigate whether including a paper questionnaire in the initial invitation would lead to a higher response rate. Furthermore, we wanted to compare patient characteristics and response rates between different modes of administration (Web-based vs paper). Our second aim was to evaluate the preference of administration mode for the older patient in particular. We hypothesized that including a paper questionnaire would increase the overall response rate because it may still be the preferred mode of administration for many older adults.

## Methods

### Setting

We used data from a large population-based survey conducted in 2010 among colorectal cancer (CRC) patients. Data were collected within the PROFILES registry [3]. The PROFILES registry collects data for the study of the physical and psychosocial impact of cancer and its treatment from a population-based cohort of short- and long-term cancer survivors. PROFILES contains a large Web-based component. However, because a large percentage of cancer patients are older, PROFILES also collects PRO data using traditional paper questionnaires. Collected PRO data are directly linked to clinical data from the Eindhoven Cancer Registry (ECR).

The questionnaire consisted of questions on work and lifestyle, health care use, comorbidity (the Self-Reported Comorbidity Questionnaire), diabetes (Problem Areas in Diabetes Questionnaire), quality of life (EORTC QLQ-C30), disease-specific symptoms (EORTC QLQ-CR38), health status (SF-12), personality (DS14), illness perception (Brief Illness Perception Questionnaire), fatigue (Fatigue Assessment Scale), and anxiety and depression (Hospital Anxiety and Depression Scale). A total of 182 items were to be answered. Respondents were informed that filling out the questionnaire could take up to 45 minutes. Online respondents could see the progress on a progress indicator and were able to log in again to continue completing the questionnaire from where they last left off. Online respondents were required to answer all questions, but could choose the option “I don’t want to say” when sensitive information was asked (eg, sexuality).

The ECR, which is part of the Comprehensive Cancer Center the Netherlands, compiles data of all newly diagnosed cancer patients in the southern part of the Netherlands, covering an area with 10 hospitals serving 2.3 million inhabitants [29]. All individuals aged between 18 and 85 years, diagnosed with CRC between January 2000 and June 2009, and registered in the ECR were eligible for participation in our study. Those with cognitive impairment, with stage 0/carcinoma in situ, and who died prior to start of the study were excluded, resulting in 3585 eligible patients. The CRC survey was approved by the Central Committee on Research Involving Human Subjects (approval number NL23463.015.08) and the Medical Ethics Committee of Máxima Medical Centre (approval number 0822). All patients signed informed consent. Further details on the method of data collection are published elsewhere [30].

### Description of Study Groups

**Figure 1:**
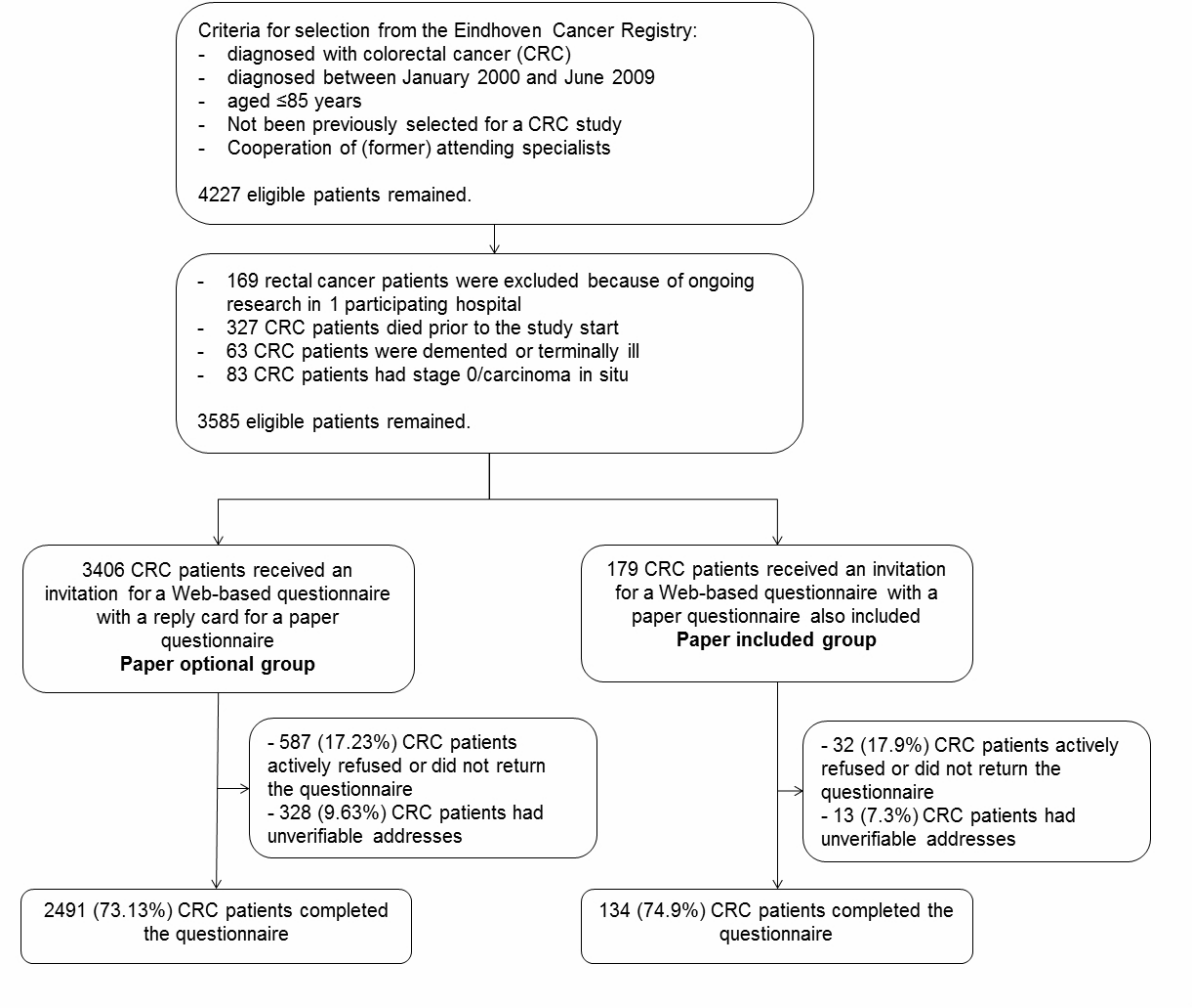
Flowchart of the colorectal cancer patient selection.

Patients were divided into 2 groups for this study (Figure 1). Patients in the paper-optional group (n=3406) were invited via a letter from their former attending specialist. The letter contained a website address and log-in instructions to fill out the Web-based questionnaire. It also contained a reply card with a return envelope (postage included) with which participants could request a paper version of the questionnaire. Patients in the paper-included group (n=179) received the same letter, but with a paper questionnaire and a return envelope (postage included) also included. Nonrespondents from both groups were sent a reminder letter together with a paper questionnaire and return envelope (postage included). A reminder was sent after 3 months, on average. The number of patients needed for the paper-included group was calculated in advance. We sampled the number of patients to be able to test a statistically significant higher (1-sided test based on our hypothesis) response rate of 10% between both groups, assuming a response rate of 75% in the paper-optional group (power 80%, alpha 10%).

### Demographics and Clinical Characteristics

Patients’ demographic and clinical information, including cancer stage, time since cancer diagnosis in years, and primary treatment were available from the ECR. The questionnaire contained questions about marital status and educational level. Information about response status, time of completion (either before or after the reminder), and the chosen mode of administration (paper or Web-based) was gathered from the PROFILES data manager application.

### Statistical Analyses

Differences in characteristics of respondents and nonrespondents and between groups were analyzed using independent *t* tests and chi-square tests where appropriate. Further analyses within the paper-optional group were conducted to assess differences in clinical and demographic characteristics between online and paper respondents. All differences with a *P* value <.05 were considered statistically significant. To assess the difference in online response between the 2 groups, logistic regression models were constructed. An unadjusted and a logistic model adjusted for age, sex, educational level, and having a partner or not were used to assess differences in Web-based response. Odds ratios (ORs) and 95% confidence intervals (95% CI) were reported. All statistical analyses were performed using SAS v9.2 for Windows (SAS institute Inc, Cary, NC, USA).

## Results

Of the 3406 invited CRC patients in the paper-optional group, 2491 (73.14%) responded. In the paper-included group, a similar response rate was found with 134 (74.9%) respondents of the 179 invited CRC patients.

Statistically significant differences in characteristics between respondents and nonrespondents were seen for gender (male: 55.16%, 1448/2625 vs 48.0%, 297/619, *P*<.001), age (mean 69.4, SD 9.53 vs mean 72.4, SD 9.9, *P*<.001), and cancer type (colon: 61.18%, 1606/2625 vs 66.9%, 414/619, *P*=.03) (Table 1). The age difference between the 2 groups was more pronounced after age was stratified into categories. The biggest response difference was found in the age category 60-70 years (32.27%, 847/2625 vs 23.4%, 145/619, *P*<.001) and ≥80 years (13.26%, 348/2625 vs 25.8%, 160/619, *P*<.001).

**Table 1:** Demographic and clinical characteristics of respondents and nonrespondents in a colorectal cancer population.

Characteristics		Respondents, n=2625		Nonrespondents, n=619		Unverifiable addresses, n=341		*P*
		Group	95% CI	Group	95% CI	Group	95% CI	
**Gender, n (%)**								
	Male	1448 (55.16)	53.26-57.06	297 (48.0)	44.0-51.9	165 (48.4)	43.1-53.7	<.001
	Female	1177 (44.84)	42.93-46.74	322 (52.0)	48.1-56.0	176 (51.6)	46.3-56.9	
Age (years), mean (SD)		69.41 (9.53)	69.05-69.78	72.4 (9.9)	71.7-73.2	68.1 (12.7)	66.8-69.5	<.001
**Age range (years), n (%)**								
	<60	410 (15.62)	14.23-17.01	65 (10.5)	8.1-12.9	80 (23.5)	18.9-28.0	<.001
	60-70	847 (32.27)	30.48-34.06	145 (23.4)	20.1-26.8	88 (25.6)	21.2-30.5	
	70-80	1020 (38.82)	37.00-40.72	249 (40.2)	36.4-44.1	109 (32.1)	27.0-36.9	
	≥80	348 (13.30)	11.96-14.55	160 (25.8)	22.4-29.3	64 (18.8)	14.6-22.9	
**Cancer type, n (%)**								
	Colon cancer	1606 (61.18)	59.32-63.05	414 (66.9)	63.2-70.6	208 (61.0)	55.8-66.1	.03
	Rectal cancer	1019 (38.82)	36.95-40.68	205 (33.1)	29.4-36.8	133 (39.0)	33.8-44.2	
Time since diagnosis (years), mean (SD)		5.16 (2.80)	5.06-5.27	5.3 (2.9)	4.9-5.4	5.5 (3.0)	5.2-5.9	.06
**Invitational approach, n (%)**								
	Paper-optional	2491 (94.90)	94.05-95.73	587 (94.8)	93.1-96.6	328 (96.2)	94.2-98.2	.57
	Paper-included	134 (5.10)	4.26-5.95	32 (5.2)	3.4-6.9	13 (3.8)	1.8-5.8	
**Mode of completion, n (%)**								
	Paper	1581 (60.23)	58.36-62.10					
	Online	1044 (39.77)	37.90-41.64					
**Time of completion, n (%)**								
	After initial request	1836 (70.16)	68.19-71.70					
	After reminder	781 (29.84)	28.00-31.50					

### Differences in Response Rates Between Groups

**Figure 2:**
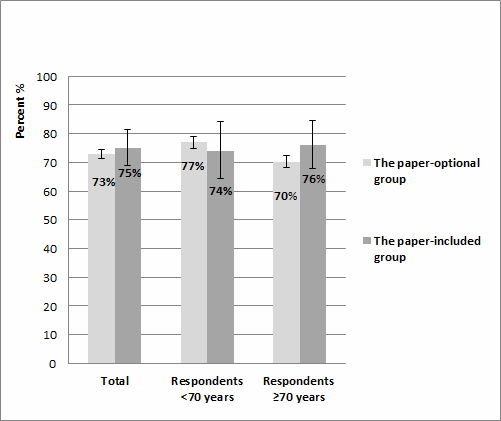
Overall response differences for the paper-optional (n=3406) and the paper-included (n=179) groups in a colorectal cancer population stratified by age groups.

No differences in overall response rate were found between the paper-optional and the paper-included groups, with 73.14% (2491/3406) and 74.9% (134/179, *P*=.57), respectively (Figure 2). For respondents aged 70 years and older, no difference in response rate was found, with a 68.84% (1290/1847) response rate in the paper-optional group and 75.7% (78/103) in the paper-included group (*P*=.38).

Characteristics of the respondents in the paper-optional group were comparable with those of the paper-included group, except for age which was slightly older in the paper-included group (≥70 years: 51.79%, 1290/2491 vs 58.2%, 78/134, *P*=.04) (Table 2). The unadjusted logistic regression model showed patients in the paper-optional group were 4.82 times (95% CI 2.88-8.07, *P*<.001) more likely to fill out the Web-based questionnaire compared to patients in the paper-included group; this effect remained after adjustments for age, sex, educational level, and having a partner or not (OR 5.81, 95% CI 3.37-10.01, *P*<.001). In the paper-optional group, online response was significantly higher compared to the paper-included group (41.23%, 1027/2491 vs 12.7%, 17/134, *P*<.001).

Sending a reminder increased the response by 30% in both arms. Due to local logistical issues, the time of sending a reminder varied between 2 and 5 months. However, this variation did not influence overall response rates (data not shown) or mean time until response (Table 2).

**Table 2:** Demographic and clinical characteristics of colorectal cancer patients in the paper-optional and the paper-included groups.

Characteristics		Paper-optional, n=2491		Paper-included, n=134		*P*
		Group	95% CI	Group	95% CI	
**Gender, n (%)**						
	Male	1368 (54.92)	52.96-56.87	80 (59.7)	51.4-68.0	.28
	Female	1123 (45.08)	43.13-47.04	54 (40.3)	32.0-48.6	
Age (years), mean (SD)		69.4 (9.59)	68.99-69.74	70.3 (8.5)	68.8-71.7	.29
**Age range (years), n (%)**						
	<60	396 (15.90)	14.46-17.33	14 (10.4)	5.3-15.6	.04
	60-70	805 (32.32)	30.48-34.15	42 (31.3)	23.5-39.2	
	70-80	954 (38.26)	36.39-40.21	66 (49.3)	40.8-57.7	
	≥80	336 (13.53)	12.15-14.83	12 (9.0)	4.1-13.8	
**Education, n (%)** ^a^						
	Low	494 (20.05))	18.27-21.40	26 (19.7)	12.7-26.1	>.99
	Medium	1488 (60.39)	57.81-61.66	80 (60.6)	51.4-68.0	
	High	482 (19.56)	17.80-20.90	26 (19.7)	12.7-26.1	
**Marital status, n (%)**						
	Married	1882 (76.19)	73.86-77.24	102 (76.7)	68.9-83.3	.23
	Divorced	136 (5.51)	4.57-6.35	8 (6.0)	2.0-10.0	
	Widow	373 (15.10)	13.57-16.38	15 (11.3)	5.9-16.5	
	Never married	79 (3.20)	2.48-3.86	8 (6.0)	2.0-10.0	
**Time since diagnosis (years), mean (SD)**		5.16 (2.80)	5.05-5.27	5.3 (2.7)	4.8-5.8	.58
Cancer type, n (%)						
	Colon cancer	1528 (61.34)	59.43-63.25	78 (58.2)	49.9-66.6	.47
	Rectum cancer	963 (38.66)	36.75-40.57	56 (41.8)	33.4-50.1	
**Mode of completion, n (%)**						
	Online	1027 (41.23)	39.30-43.16	17 (12.7)	7.1-18.3	<.001
	Paper	1464 (58.77)	56.84-60.70	117 (87.3)	81.7-93.0	
**Time of completion, n (%)**						
	After first invitation	1743 (70.14)	68.17-71.77	93 (70.5)	61.6-77.2	.94
	After reminder	742 (29.86)	27.99-31.58	39 (29.5)	21.4-36.8	
**Time until response (days), mean (SD)**						
	After first invitation	21.46 (19.32)	20.56-22.37	19.1 (12.1)	16.6-21.6	.23
	After reminder	20.11 (15.24)	19.01-21.21	19.4 (15.0)	14.5-24.2	.70

^a^ Low: no/primary school; medium: lower general secondary education/vocational training; high: preuniversity education/high vocational training/university.

### Comparison of Characteristics of Online and Paper Respondents in the Paper-Optional Group With a Focus on Older Patients

In the paper-optional group, men were more likely to complete the Web-based questionnaire than to return the paper questionnaire (61.73%, 634/1027 vs 50.14%, 734/1464, *P*<.001) (Table 3). Compared to paper respondents, online respondents were younger (mean 65.72, SD 9.28 vs mean 71.85, SD 8.89, *P*<.001), more often highly educated (30.34%, 311/1027 vs 11.88%, 171/1464, *P*<.001), more often married (83.93%, 862/1027 vs 70.69%, 1020/1464, *P*<.001), more often recently diagnosed (time since diagnosis: mean 4.94, SD 2.74 vs mean 5.31, SD 2.83, *P*<.001), and more often had a rectal cancer diagnosis compared to paper respondents (41.38%, 425/1027 vs 36.75%, 538/1464, *P*=.02). The majority of the online respondents responded after the first invitation (95.42%, 979/1027), which was significantly higher than the paper respondents (52.36%, 764/1464, *P*<.001).

**Table 3:** Demographic and clinical characteristics of colorectal cancer patients in the paper-optional group stratified by questionnaire type used.

Characteristics		Paper questionnaire, n=1464		Web-based questionnaire, n=1027		*P*
		Group	95% CI	Group	95% CI	
**Gender, n (%)**						
	Male	734 (50.14)	47.58-52.70	634 (61.73)	58.76-64.71	<.001
	Female	730 (49.86)	47.30-52.42	393 (38.27)	35.29-41.24	
Age in years, mean (SD)		71.85 (8.89)	71.41-72.29	65.72 (9.28)	65.16-66.29	<.001
**Age range (years), n (%)**						
	<60	150 (10.25)	8.70-11.80	246 (23.95)	21.34-26.56	<.001
	60-70	362 (24.73)	22.52-26.94	443 (43.14)	40.11-46.16	
	70-80	677 (46.17)	43.69-48.80	277 (26.97)	24.26-29.69	
	≥80	275 (18.85)	16.78-20.78	61 (5.94)	4.49-7.39	
**Education, n (%)** ^a^						
	Low	400 (27.80)	25.04-29.61	94 (9.17)	7.39-10.92	<.001
	Medium	868 (60.32)	56.77-61.81	620 (60.49)	57.38-63.36	
	High	171 (11.88)	10.04-13.32	311 (30.34)	27.47-33.09	
**Marital status, n (%)**						
	Married	1020 (70.69)	67.32-72.03	862 (83.93)	81.69-86.18	<.001
	Divorced	82 (5.68)	4.42-6.78	54 (5.26)	3.89-6.62	
	Widow	288 (19.96)	17.64-21.71	85 (8.28)	6.59-9.96	
	Never married	53 (3.67)	2.66-4.58	26 (2.53)	1.57-3.49	
**Time since diagnosis (years), mean (SD)**		5.31 (2.83)	5.17-5.45	4.94 (2.74)	4.77-5.11	<.001
Cancer type, n (%)						
	Colon cancer	926 (63.25)	60.78-65.72	602 (58.62)	55.61-61.63	.02
	Rectal cancer	538 (36.75)	34.28-39.22	425 (41.38)	38.37-44.39	
**Time of completion, n (%)**						
	After initial request	764 (52.36)	49.63-54.74	979 (95.42)	94.04-96.62	<.001
	After reminder	695 (47.64)	44.91-50.03	47 (4.58)	3.30-5.85	
**Time until response (days), mean (SD)**						
	After first invitation	36.08 (17.05)	34.87-37.29	10.03 (11.84)	9.29-10.77	<.001
	After reminder	20.66 (15.21)	19.53-21.80	11.76 (13.34)	7.80-15.72	<.001

^a^ Low=no/primary school; medium=lower general secondary education/vocational training; or high=preuniversity education/high vocational training/university.

**Figure 3:**
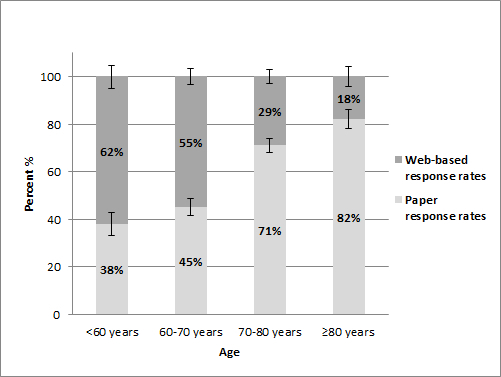
Web-based and paper response rates in the paper-optional group among colorectal cancer patients stratified by age groups (n=2491).

After age was stratified, Web-based versus paper response differed per age group (*P*<.001, Figure 3). We saw that the turning point of filling out a Web-based questionnaire was approximately age 70 years: the majority of respondents younger than 70 years filled out a Web-based questionnaire and the majority older than 70 years chose a paper questionnaire. Among those aged ≥80 years, 18.2% (61/336) preferred a Web-based questionnaire.

## Discussion

### Principal Findings

Our study showed that including a paper questionnaire with the first invitation did not increase overall response rates. In contrast, it negatively influenced the online response. Sending a reminder improves response rates by 30%. Compared to patients responding on paper, online respondents were more often male, younger, married, and highly educated. The majority of respondents in both arms chose to fill out a paper questionnaire. The turning point of preference for a Web-based questionnaire was approximately age 70 years. The majority of respondents who were younger than 70 years preferred to fill out the Web-based questionnaire. The majority of respondents older than 70 years chose the paper questionnaire. We did not find evidence that including a paper questionnaire led to a higher response among older patients.

We expected the overall response rate in the paper-included group to be higher than in the paper-optional group because respondents in the paper-included group received the paper questionnaire immediately at invitation. However, we observed similar overall response rates. A previous review of the literature showed that when respondents can choose between paper and Web-based questionnaires, paper response is higher than online response in most studies [31]. A recent literature review confirmed this, although they expect the difference to diminish in the near future [6]. The absence of this expected difference in response rates in our study could not be explained by differences in patient characteristics between the 2 groups. A possible explanation for the comparable response rates could be the willingness of respondents to participate because the subject of the questionnaire (ie, cancer and health-related quality of life) felt relevant to them. Furthermore, the respondents received the invitation directly from their medical specialist, so they might have felt a moral obligation to participate. The lack of difference in response rates implies that researchers can leave out a questionnaire at first invitation without lowering response. It is preferable not to include a paper questionnaire because more respondents will fill out the Web-based version of a questionnaire, which will enable researchers to access data more quickly and to have a more complete dataset.

Several studies have compared response rates between patients invited via paper only and Web only, or mixed-mode and Web only, or paper only and mixed-mode [5,7,8,10,11,13,14,32-36]. However, few studies are available that address the influence of including a paper questionnaire on response rate in the invitation for a mixed-mode survey. We found an American study that compared the response rates of 3 modes of administration, namely paper only, paper with an Internet option, or Internet with a paper option [37]. The response for the Internet with a paper option and for the paper with an Internet option was 37% and 42%, respectively. These are the same manners of invitation we used in our study. The difference with our study is that instead of sending 1 reminder, the other study sent 4 reminders. Only the last reminder for the Internet with a paper option contained a paper questionnaire. A second difference is that this study was done in 2000 in the United States, so the use of Internet was lower than in 2010 in the Netherlands, when our study was done. Internet use in the United States in 2000 was 51% compared to 90% in the Netherlands in 2010 [38,39]. This might explain the lower response rates for both groups and the bigger difference in response rates between the groups in that study. A Dutch study among 277 long-term childhood cancer survivors in 2010 used a comparable invitation approach and mode of administration [40]. The study used a mixed invitation group (paper with the option of Internet) and a Web-only invitation group (Internet with the option of paper) leading to a response of 83% and 89%, respectively. A different approach with regard to reminders was chosen in that study compared to ours; after sending 1 reminder letter, nonrespondents were contacted by telephone in their study. Another difference is that only young women were included in that study. Both studies [37,40] did not address the (preference of) the older patient.

When studying different age groups, we found that almost 20% of the respondents aged ≥80 years filled out the questionnaire online. We expected a lower percentage because of the so-called “grey digital divide” referring to the low use of computers and the Internet in the older population [41]. This grey digital divide is also confirmed by a British study that found that only 10% of respondents aged ≥85 years have used the Internet at any point in their lives [22]. To fill out a Web-based questionnaire, a respondent must not only be able to use a computer, but also be skilled on the Internet. The high number of older respondents who used the Internet in our study might imply that the grey digital divide is closing in the Netherlands and more older people are becoming familiar with the Internet. Numbers from Statistics Netherlands (CBS) confirm this, showing that there is an increase in Internet use among individuals aged between 65 and 75 years in recent years [28]. Daily use of the Internet among these individuals increased from 15% in 2005 to 55% in 2013. Eurostat Statistics also show these numbers: a rise in frequent use of the Internet among people aged 65 to 74 years from 41% in 2008 to 73% in 2013 in the Netherlands [42]. Unfortunately, a group of users is left out in these statistics, namely the people older than 75 years. The statistics do, however, indicate a trend of older people being more online. With this in mind, researchers could more easily consider using the Internet as a primary mode for data collection without the inclusion of a paper questionnaire with the first invitation.

Strengths of this study are that it is population-based, including (very) older people, has a high overall response rate, and the cooperation of medical specialists. Furthermore, the influence of sending a paper questionnaire in 2 mixed-mode groups has rarely been studied. Thirdly, our results are more recent than other studies that compare paper versus Web-based questionnaires, which is important because of the rapid changes in Internet access. Lastly, we have looked at many patient characteristics to assess the differences in patient characteristics of online and paper respondents.

A limitation of this study is that the time a reminder was sent varied per hospital due to local logistical issues. However, analyses showed that the difference in reminder time did not have any effect on outcomes. A second limitation is that the comparison between the paper-optional group and the paper-included group shows a slight discrepancy in the age categories, although mean age did not differ. There is a slightly higher percentage of respondents older than 70 years in the paper-included group. Although an age difference existed before data collection in the initial random selection of this group, it was not significant (results not shown). Thus, the significant discrepancy found in our results after data collection is a consequence of (un)willingness to cooperate. In the future, further evaluation of nonrespondents may clarify this difference. It is not unlikely that the results found in our study are applicable to other populations, for example, patients with a different type of cancer, a different disease (eg, diabetes), or a normative population. However, further research is needed to confirm this.

### Conclusion

Although this study was on a CRC survivor population, we are of the opinion that the significant lack of difference in response rates between invitation modes implies that researchers may leave out a paper questionnaire at invitation without lowering the response rate. It may even be more preferable not to include a paper questionnaire because more respondents then will fill out a questionnaire online, which will lead to faster available data. However, due to respondent preference, it is not likely that paper questionnaires can be left out completely in the near future.

## Abbreviations

CRC: colorectal cancer
ECR: Eindhoven Cancer Registry
PRO: patient-reported outcomes
PROFILES: Patient-Reported Outcomes Following Initial treatment and Long-Term Evaluation of Survivorship
